# Diagnosis and management of extrahepatic oesophageal variceal bleed in children in a low resourced setting

**DOI:** 10.4314/gmj.v54i4.11

**Published:** 2020-12

**Authors:** Taiba J Afaa, Kokou H Amegan-Aho, Elikem Richardson, Bamenla Goka

**Affiliations:** 1 Department of Child Health, University of Ghana Medical School, College of Health Sciences, University of Ghana, Accra; 2 Department of Child Health, Korle Bu Teaching Hospital, Accra; 3 Department of Paediatrics and Child Health, School of Medicine, University of Health and Allied Sciences, Ho, Ghana

**Keywords:** Variceal bleed, extrahepatic portal vein obstruction, children, oesophageal varices

## Abstract

Extrahepatic portal vein obstruction (EHPVO) is a major cause of portal hypertension (PH) in children. Portal vein thrombosis (PVT) is the most common cause accounting for up to 75% of cases in developing countries. Upper gastrointestinal bleeding is the most dreaded and commonest presentation of portal hypertension. Successful treatment of paediatric PH, though challenging is performed in resource constraint countries.

**Cases:**

Five children presented over three years to a tertiary hospital in Ghana, with massive upper gastrointestinal bleeding. They had anaemia, thrombocytopaenia and four had splenomegaly. Liver function tests, INR, haemoglobin electrophoresis as well as HIV serology, hepatitis B and C screening were all normal. Abdominal doppler ultrasound scan confirmed portal vein thromboses. They were resuscitated and managed with octreotide, propranolol, antibiotics and sclerotherapy or oesophageal variceal banding in the acute setting and long term secondary prophylaxis with propranolol. Subsequently, an algorithm was developed to assist with the management of bleeding from oesophageal varices and the diagnosis of EHPVO.

**Conclusion:**

Portal hypertension due to EHPVO is an important cause of upper gastrointestinal (GI) bleeding in children. This can be successfully managed even in a resource constraint setting once the appropriate measures are taken.

## Introduction

Extrahepatic portal vein obstruction (EHPVO) is a major cause of portal hypertension (PH) in children. Portal vein thrombosis (PVT) is the most common cause accounting for up to 75% of cases in developing countries.^1^,^2^ The portal system supplies the liver with nutrient rich and partially oxygenated blood to augment the highly oxygenated blood from the hepatic artery.^3^ It has a low baseline pressure of 7 mm Hg to 10 mm Hg and PH occurs when the pressure exceeds 10 mm Hg. The 4 main causes of EHPVO are perinatal events (umbilical catheterization, omphalitis, and dehydration)^4^, extrahepatic porto-caval shunts,^3^ hereditary (deficiency of protein-C, S or antithrombin-III, Factor V Leiden mutation) and acquired thrombophilia (tumours, abdominal infections)5, and idiopathic causes that account for more than 50% of cases.^6^

Portal hypertension commonly presents with upper gastrointestinal (GI) bleeding, due to variceal rupture.^7^ Oesophageal varices are present in 90% to 95% and gastric varices in 35% to 40% of patients with PH.^8^ Mortality is up to 48% within the first 3 days of bleeding.^9^ Splenomegaly is the second major clinical manifestation of PH. Other features include poor growth (50%), ascites in advanced cases and biliary changes. Laboratory findings include anaemia, leucopoenia and thrombocytopenia due to hypersplenism. Patients with EHPVO have normal or near normal liver function and coagulation profile. Doppler ultrasonography is the most useful diagnostic tool for the diagnosis of PH and EHPVO, with a sensitivity and specificity above 95%.^11^ Additionally, contrast-enhanced computed tomography or magnetic resonance angiography are useful for assessing the extent of obstruction and can serve as a road map if surgery is needed.^12^

Treatment for PH includes endoscopic treatment (sclerotherapy or banding), pharmacologic therapy to reduce portal pressures, and surgery. We present five children seen in Korle Bu Teaching Hospital (KBTH) with EHPVO who were successfully resuscitated and treated with pharmacologic and endoscopic therapies.

## Case Reports

These patients were referred to the KBTH between March 2017 to January 2020 with upper GI bleeding. Liver function tests, INR, haemoglobin (Hb) electrophoresis as well as HIV serology, hepatitis B and C screening were all normal. Full blood counts and abdominal ultrasound scan were carried out for all the children. Table 1 summarises the clinical features and treatment of the patients.

**Table 1:** Summary of the clinical features and treatment modalities for patients with variceal bleeding

Patient	1	2	3	4	5
**Age(years)**	7	4	9	14	10
**Sex**	Male	Male	Female	Female	Male
**Duration since first** **bleed**	5 days	4 days	4 years	18 months	2 years
**Neonatal events**	Normal	Normal	Preterm	Preterm	Normal
**Presentation**	Massive	Haematemesis	Recurrent	Recurrent	Recurrent
	haematemesis	Melaena	haematemesis	haematemesis	haematemesis
		Fever	Melaena, fever		Melaena, fever
**Clinical features**	Splenomegaly	Hepatomegaly	Splenomegaly	Splenomegaly	Splenomegaly
	Underweight	Underweight	Low platelets	Low platelets	Low platelets
	stunted	Low platelets			
	Low platelets				
**Treatments**	Blood transfusion	Blood transfusion	Blood transfusion	Prednisolone	Blood transfusion
	Propranolol	PPI	PPI	Carvedilol	PPI
	Antibiotic	Tranexamic	Tranexamic acid	EVL	Tranexamic
	Octreotide	acid	Vitamin K		acid
	EVL	Vitamin K	Omeprazole		Antibiotic
		Propranolol	Antibiotic		Octreotide
		Antibiotic	Octreotide		EVL
		Octreotide	EST		
		EST			
PPI: *proton pump* *inhibitor*					

EST: *Endoscopic sclerotherapy*; EVL: *Endoscopic variceal band ligation*

### Case 1

A seven-year-old male was referred with a first episode of massive haematemesis. He was transfused with 3 pints of blood at the referral site without much improvement. He had a normal neonatal period. On examination, he was stunted and wasted, very pale, not jaundiced, not clubbed, afebrile and had splenomegaly of 6 cm. His Hb was 3.6g/dL, white cell count (WBC) 8.6x10^9^/L and platelets 79 × 10^9^/L. He was started on intravenous (IV) normal saline, subcutaneous octreotide at 3 mcg/kg/8 hourly, IV ciprofloxacin and transfused one unit of blood. His bleeding stopped within 2 days. Upper GI endoscopy showed Grade 3 varices, which were managed with four sessions of endoscopic variceal band ligation (EVL). He received maintenance oral propranolol at 1mg/kg/day in three divided doses and this was stopped after the eradication of the varices. He comes annually for surveillance endoscopy.

### Case 2

A four-year-old male presented with 4 days history of haematemesis and passage of melaena stools. His Hb at presentation, after receiving a pint of blood, was 4.5g/dL. He had a normal neonatal period. He was febrile (38.2°C), with hepatomegaly of 2cm and no splenomegaly. The Hb improved to 6.8 g/dL after the second blood transfusion, with WBC of 12.6 x10^9^/L and thrombocytopenia of 120 × 10^9^/L. He was given tranexamic acid, vitamin K and antibiotics, but the bleeding continued. Tranexamic acid and vitamin K were stopped, and octreotide was introduced which dramatically stopped the bleeding. Doppler ultrasound showed echogenic lesions in the region of the portal vein, with no spontaneous flow or evidence of recanalization. Upper GI endoscopy done showed Grade 3 oesophageal varices and a positive *Helicobacter pylori* test, which was treated with triple therapy. Oesophageal varices were managed with six sessions of endoscopic sclerotherapy (EST) using 5% ethanolamine oleate and maintenance oral propranolol at 1mg/kg/day in three divided doses. He is due for surveillance endoscopy after a year of follow up without recurrence of bleeding.

### Case 3

A nine-year-old female presented with the fifth episode of massive haematemesis over a 4-year period. Each of the four previous episodes was managed with blood transfusion at the district hospital and she was discharged home. She was delivered pre-term at 28 weeks gestation and she was admitted at the neonatal ward for 6 weeks. Mother could not recall if her daughter had had an umbilical catheterisation whilst on admission. There was a history of recurrent jaundice, gum bleeding, palpitations, easy fatigability, dizziness and use of oral herbal medications. She looked acutely ill and severely pale with no stigmata of chronic liver disease. She had a mild epigastric tenderness, splenomegaly of 3cm and dark starry stool on digital rectal examination. The was Hb 5.9g/dL (post-transfusion), WBC of 12.03 × 10^9^/L with relative neutrophilia and thrombocytopaenia of 141 x 10^9^/L. A blood film comment showed normocytic normochromic red cells with markedly reduced platelets. She received IV tranexamic, vitamin K, IV omeprazole and 5 pints of blood on admission. Upper GI endoscopy showed tumour-like Grade 3 oesophageal varices with portal hypertensive gastropathy. A doppler ultrasound confirmed cavernous transformation of portal vein due to chronic PVT. Oesophageal varices were treated with sclerotherapy using 99.9% ethanol. Two days later, she had another episode of haematemesis, which was managed with octreotide and antibiotics. The bleeding resolved in two days. She had a total of six sessions of sclerotherapy, which led to the eradication of the oesophageal varices.

### Case 4

A14-year-old female child presented with a second episode of hematemesis within 18 months and was initially managed by haematologists with a diagnosis of immune thrombocytopenic purpura. She was born premature at 32 weeks gestation and was admitted for two months. There was no history of umbilical catheterisation. The platelet level had improved on steroids with no further bleeding episodes. She was later referred to surgery for splenectomy, but the surgeons suspected PH and requested for upper GI endoscopy, which revealed oesophageal varices. Abdominal doppler ultrasound confirmed PVT. She was started on carvedilol at 3.125 mg twice daily and the varices were managed with EVL. She did not have any further episodes of bleeding.

### Case 5

A10-year-old male presented with a fifth episode of haematemesis and melaena over a period of two years for which he had had several blood transfusions, IV tranexamic acid, lactulose and oral metronidazole at peripheral hospitals. The last episode prompted referral to KBTH on account of syncope. He was delivered at term with normal neonatal period. He had had herbal medication intermittently. His Hb was 5.9 g/dL and he was transfused with whole blood. He was anicteric, febrile (39.6°C) with splenomegaly of 6 cm. The platelet count was 115 × 10^9^/L. The acute bleeding improved with octreotide and antibiotics. Abdominal doppler ultrasound showed cavernous malformation in the region of the portal vein secondary to chronic thrombus. He was managed successfully with the oesophageal varices were managed with EVL for oesophageal varices.

## Discussion

All five patients presented with upper GI bleed, the commonest feature of PH, which has a high mortality if not properly managed.^12^ Four patients had splenomegaly with thrombocytopaenia and two were underweight. Growth faltering is linked to reduced portal blood supply to the liver resulting in reduced production of insulin-like growth factor that regulate liver function and physical growth.^7^ When splenomegaly is found incidentally in any child together with thrombocytopaenia and or leucopoenia, haematological and oncological causes must be ruled out first. After that, PH should be considered. Abdominal ultrasound will show anatomic abnormalities but not vascular problems. As seen in the patients above, abdominal doppler ultrasound should be requested in addition to abdominal ultrasound in suspected variceal bleeding so that, EHPVO can be confirmed or excluded as the underlying cause. Most of the modalities of management of paediatric PH are based on adult trials.^7^

Only 20% (two patients) had neonatal events that can be attributed to the cause of the PVT. This is similar to what Karaksy et al found in Egyptian children.^13^ Worldwide only 50% of children have a known cause of their PVT.^7^ Upper GI bleed, which is the dreaded presentation of PH, will spontaneously stop in 40 to 50% 14 of cases but rebleeding is common, hence definitive therapy is required. Immediate resuscitation with intravenous crystalloids and blood transfusion should restore haemoglobin levels to about 8 g/dL^5^ and ensure good perfusion of vital organs while monitoring vital signs closely. Overzealous use of volume/plasma expanders should be avoided, however, because of the risk of rebound PH and rebleeding.^15^ Placement of a nasogastric tube allows monitoring for on-going bleeding as well as removing the blood from the GI tract to reduce the likelihood of vomiting as blood is an irritant to the gastric mucosa. All patients with confirmed or suspected variceal bleeding should be started on splanchnic vasoconstrictor agents like octreotide as early as possible to reduce acute bleeding, the need for blood transfusion and mortality while promoting easier and safer endoscopic procedures.^16^ The dose of octreotide is 1–5 µg/Kg by bolus over 20 minutes, followed by continuous infusion at 2 µg/Kg/h and should be continued for 2–5 days.^16^ Octreotide can also be given at the same dose eight hourly via the subcutaneous route. Tranexamic acid slows the breakdown of blood clot by inhibiting the action of plasmin, which is involved in fibrinolysis.^17^ Its use in three of our patients did not control the bleeding as this was from ruptured high-pressure oesophageal varices. The use of tranexamic acid in the control of variceal bleeding is not recommended. Variceal bleeding is often preceded by a febrile illness although only two of our patients presented with fever. Systemic bacterial infection is an independent risk factor for variceal rupture. Antibiotics directed at pathogenic intestinal flora should be started in all patients with suspected or confirmed variceal bleeding to reduce bacterial infection, recurrent bleeding, and mortality.^18^

Once the patient is stabilized, upper GI endoscopy is used to confirm the presence and provide treatment of the varices. The two types of endoscopic therapies are EST and EVL. Both are highly effective in controlling acute variceal bleeding in over 90% of cases as well as in eradication of varices.^12^ EST has also been shown to be useful even in very small children as young as 5 months weighing 5.5 kg.^7^ The most widely used sclerosant is ethanolamine, in the absence of which, we used 99.9% ethanol for one of the patients with good results.^19^ In between periods of endoscopic therapy, a nonselective beta-blocker (propranolol)^20^ is used to reduce hepatic venous pressure gradient by decreasing cardiac output (beta1-receptor antagonism) and inducing splanchnic vasoconstriction (beta2- receptor antagonism) thereby reducing the portal pressure. Carvedilol was used in one of the patients who responded quiet well. A systematic review and metaanalysis of 12 RCTs showed that carvedilol is more effective in decreasing hepatic venous pressure than propranolol and with fewer side side effects.^21^ Based on the success of the cases treated above, an algorithm has been developed to assist doctors in the country and the sub region to diagnose and manage portal hypertensive variceal bleed due to EHPVO (Annex 1).

**Annex 1:**
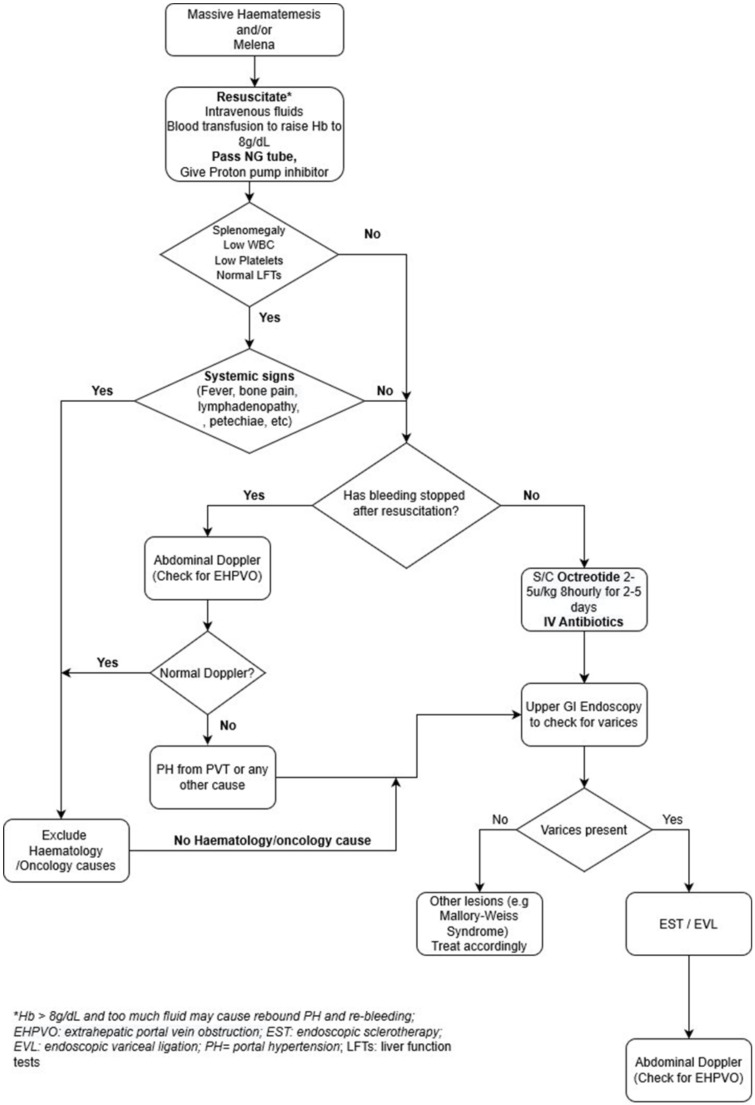
Algorithm for the diagnosis and treatment of variceal bleed from extrahepatic portal vein obstruction in children.

## Conclusion

Portal hypertension due to EHPVO is an important cause of upper GI bleeding in children. This can be successfully managed even in a resource constraint setting once the appropriate measures are taken.
